# Cerebral visual impairment is permanent but not unchanging

**DOI:** 10.1111/dmcn.16137

**Published:** 2024-10-26

**Authors:** Cathy Williams

**Affiliations:** ^1^ Bristol Medical School University of Bristol Bristol UK

## Abstract

This commentary is on the original article by Galli et al. on pages 486–495 of this issue.

The study of cerebral visual impairment (CVI) is still an emerging field, with ongoing uncertainties about diagnosis, assessment, and the effects of intervention. Galli et al. contribute one of the few natural history studies relating to CVI.^1^ Their longitudinal study documents visual problems (including CVI) in a group of children with cerebral palsy, followed from infancy/early preschool into their school years. They found that some manifestations may improve, some remain constant, and some worsen or only become evident as the child matures. CVI is thus ‘permanent but not unchanging’.

The paper by Galli et al. is helpful for several reasons. First, with these data we can give parents and their children reliable advice about how their CVI might change over time, as the results are unbiased by variations in sampling that affect cross‐sectional studies. There are improvements in visual acuity for many (as in typically developing children) and in oculomotor control for some, as well as warnings regarding the development of later visual problems.

Second and importantly, the authors show that some clinical signs – disturbances in pursuit eye movements – elicited in the very early years predicted later cognitive visual disorders (CVDs). This potential early warning sign means children can be provided with appropriate support as soon as they start to struggle (if they do) and prioritized for neuropsychological or educational psychological assessments to look for CVDs when they are old enough, rather than waiting for them to fail over time and develop entrenched difficulties. It also adds to the rationale for early therapeutic interventions at a time when neuroplasticity is greatest for at‐risk children.

Third, the authors show how the simple but rigorously conducted tests in their ‘neurovision’ protocol (involving assessments for ophthalmic, oculomotor, basic visual function, and CVDs) can be used at different ages. The protocol is firmly grounded in developmental visual neurophysiology and can diagnose CVI if present along with other visual problems, for example refractive errors and strabismus. The inclusion of optic nerve characteristics is important as retrograde degeneration of neurons connected to damaged areas in the central visual pathways may take several years to be detected by ocular examination and/or retinal scans.^2^ As the authors point out, anticipating these developments avoids children having unnecessary tests to investigate optic nerve problems. Protocols like this allow a holistic approach to the child's overall visual profile, capturing their difficulties in multiple visual domains.

Galli et al. report that all their participating children were from upper middle‐class families and the majority (86%) were white. However, we know that the highest prevalence of children with certifiable visual impairment in the UK (of which approximately half is due to CVI) is reported in children from the most socioeconomically deprived families and in ethnicities other than white.^3^ Certainly one risk factor for CVI – premature birth – is reported as more frequent for infants born in socially disadvantaged areas in the USA than for infants born in more affluent areas;^4^ and several socially determined risk factors such as poor maternal nutrition and smoking are associated with higher risks of preterm birth across European countries.^5^

By comparing CVI profiles between timepoints in individuals, Galli et al. have added to our ability to counsel families of children with CVI and cerebral palsy, and to take advantage of early warning signs to try and reduce the impact of CVI. Using standard protocols like this, and consistently reporting participants' demographic details, could also help us to compare CVI profiles between children from different backgrounds and further develop the evidence base with which we can improve outcomes for all children with CVI.

## Data Availability

Not required.
